# Bilateral visual acuity decline in males with choroideremia: a pooled, cross-sectional meta-analysis

**DOI:** 10.1186/s12886-022-02250-z

**Published:** 2022-01-16

**Authors:** Duygu Bozkaya, Heng Zou, Cindy Lu, Nicole W. Tsao, Byron L. Lam

**Affiliations:** 1 Biogen, Cambridge, MA USA; 2 Bascom Palmer Eye Institute, Miami, FL USA

**Keywords:** Visual acuity, Retinal disease, Eye symmetry, Bilateral, Interocular

## Abstract

**Background:**

Choroideremia is a rare inherited retinal disease that leads to blindness. Visual acuity (VA) is a key outcome measure in choroideremia treatment studies, but VA decline rates change with age. An accurate understanding of the natural deterioration of VA in choroideremia is important to assess the treatment effect of new therapies in which VA is the primary outcome measure. We conducted a meta-analysis of data on individuals with choroideremia to determine the rate of VA deterioration between the better- and worse-seeing eye (BSE and WSE, respectively).

**Methods:**

Data were collected from the prospective Natural History of the Progression of Choroideremia (NIGHT) study (613 eyes, baseline data only), studies included in a recent meta-analysis, and studies identified in a targeted literature search performed on March 25, 2020, including individual best-corrected VA (BCVA) and age data in male individuals with choroideremia. Best-corrected VA decline rates (measured by logMAR units) by age and trends in BCVA decline rates in the BSE and WSE were evaluated.

**Results:**

Data from 1037 males (1602 eyes; mean age, 41.8 years) were included. Before and after an age cutoff of 33.8 years, BCVA decline rates for the WSE were 0.0086 and 0.0219 logMAR per year, respectively. Before and after an age cutoff of 39.1 years, BCVA decline rates for the BSE were 0.00001 and 0.0203 logMAR per year, respectively. Differences in absolute BCVA and decline rates increased between the 2 eyes until age ~ 40; thereafter, differences in absolute BCVA and decline rates were similar between eyes.

**Conclusions:**

Using the largest choroideremia data set to date, this analysis demonstrates accelerated BCVA decline beginning between 30 and 40 years of age. Disparate interocular progression rates were observed before the transition age, with similar interocular progression rates after the transition age.

**Supplementary Information:**

The online version contains supplementary material available at 10.1186/s12886-022-02250-z.

## Background

Choroideremia is a rare inherited retinal disease that results in progressive vision loss, ultimately leading to blindness [1–7]. Symptoms often first appear in early childhood or adolescence as night vision impairment, followed by a progressive decline in peripheral vision. Vision impairment continues to worsen, resulting in loss of central visual function and legal blindness, which occurs at a median age of 66 to 72 years according to a recent meta-analysis [8]. The progressive visual impairment in choroideremia can contribute to worsening functional and emotional burdens, including loss of independence; difficulties in performing daily activities; diminished quality of life; and increased stress, anxiety, or depression [9–13]. Choroideremia is caused by mutations in the *CHM* gene on the X chromosome, leading to predominant occurrence in males [14, 15]. Because of overlapping symptoms with other retinal diseases, choroideremia may be underdiagnosed, and more recognition of the natural progression of choroideremia may facilitate diagnosis.

Although there are no approved choroideremia treatments, investigational gene therapies are being evaluated in clinical studies [16–18]. As clinical studies for choroideremia include visual acuity (VA) as an outcome [19–21], it is important to understand and establish an accurate description of the natural deterioration of VA in choroideremia to assess the treatment effect that new therapies may have in changing the disease course. Previous studies investigating the natural decline of best-corrected VA (BCVA) in choroideremia suggest an accelerated BCVA decline between the ages of 30 to 60 years [2, 4, 5, 7, 22–24]. The transition age of accelerated BCVA decline as well as the rate of BCVA decline before and after the transition age varied among studies; this variability could be attributed to the differing sample sizes and statistical methods used. In a meta-analysis reporting on data from 1004 eyes, Shen et al. addressed these points [8]. The results of the meta-analysis supported a 2-phase linear model of BCVA decline with a transition age of 39 years.

Shen et al. also evaluated rates of BCVA decline in the worse-seeing eye (WSE) and better-seeing eye (BSE) and determined that the median ages of impaired VA, subnormal VA, and legal blindness occurred earlier in the WSE compared with the BSE. The objective of the present study was to perform an updated meta-analysis incorporating additional data from 613 eyes from the prospective Natural History of the Progression of Choroideremia (NIGHT) study to further evaluate the progression of BCVA decline and rate of decline in the WSE and BSE in choroideremia.

## Methods

### Eligibility criteria and study identification

This meta-analysis includes published studies reporting BCVA data of individuals with choroideremia and baseline data from the NIGHT study, which reported longitudinal BCVA data for both eyes for all participants. Rates of BCVA decline according to age and rates of BCVA decline between the WSE and BSE were evaluated. All articles included in the current analysis were initially identified from the systematic literature review by Shen et al. [8]. Data from Aleman et al. [7] and Dysli et al. [25] were excluded because data could not be extracted from the graphs or individual-level BCVA measurements by age could not be identified. A nonsystematic targeted literature review was conducted with the goal of identifying newly published articles or relevant articles not reviewed by Shen et al. Searches were conducted in PubMed® (National Library of Medicine, Bethesda, MD), Embase® (Elsevier, Amsterdam, Netherlands), and Web of Science™ (Clarivate, London, United Kingdom) on March 25, 2020. The literature search was performed by one individual, and all articles were reviewed for inclusion in the analysis by the authors. The search strings used were “choroideremia AND visual acuity” and “choroideremia AND disease progression AND age” without date limits. Articles included were written in English or Chinese; Chinese articles were reviewed by a native speaker. Articles reporting individual-level BCVA data by age either cross-sectionally or longitudinally were included. Data from females were excluded because of limited sample size and previously reported differential VA deterioration patterns for female carriers compared with male individuals [24]. The number of individuals and available data points from studies in the literature reported in this meta-analysis may vary slightly from those reported in the original studies because, in several instances, some data were extracted from graphs in which an individual may have contributed to the graph more than once, a data point on the graph may have represented more than one individual, or an individual was included in more than one study.

The prospective, global, multicenter NIGHT study is the largest natural history study of choroideremia (ClinicalTrials.gov identifier, NCT03359551) [3, 26–29]. Research Ethics Committee (Independent Ethics Committee or Institutional Review Board) and host institution approval were obtained. Males aged ≥18 years with a genetically confirmed diagnosis of choroideremia, active disease within the macula, and BCVA Early Treatment Diabetic Retinopathy Study (ETDRS) letter score of > 33 (20/200, 1.0 logMAR) in at least 1 eye were eligible for the study. Participants attended 6 visits over a 20-month period (1 visit every 4 months), during which comprehensive functional and anatomical ophthalmic assessments were performed, including BCVA, which was measured using the ETDRS protocol [3, 28, 29]. Baseline BCVA data were used in the current analysis.

### Statistical analyses

Descriptive statistics were performed for BCVA at age groups categorized by < 10, 10 to < 20, 20 to < 30, 30 to < 40, 40 to < 50, 50 to < 60, 60 to < 70, 70 to < 80, 80 to < 90, and 90 to < 100 years. Best-corrected VA was reported in logMAR units, a measure recognized as being more reliable and discriminative of interocular differences compared with Snellen equivalent [30]. Categories for BCVA were based on the definition by the International Council of Ophthalmology: normal/near normal vision (BCVA of ≤0.5), moderate-to-severe visual impairment (BCVA of > 0.5 to ≤1.3), profound visual impairment (BCVA of > 1.3 to ≤1.7), and blindness/near blindness (BCVA of > 1.7) [31]. A segmented regression model was fit to the linear relationship between BCVA and age before and after specific cutoff points to define distinct decline rates. Trends in interocular disease progression were assessed by evaluating differences between the BSE and WSE as well as coefficient correlations between the 2 eyes in individuals with available bilateral data. Age cutoffs for transition age in the BSE, WSE, and both eyes were determined using segmented regression. All analyses were performed in SAS version 9.4. Sensitivity analyses were performed by excluding data from the NIGHT study.

## Results

### Sample population and age

Overall, 23 studies were identified encompassing 717 individuals and 989 eyes (see Supplementary Fig. 1 and Supplementary Table 1 in Additional File 1) [2, 4, 5, 20, 22, 23, 32–48]. All included studies were identified in a recent meta-analysis [8]. Among the additional relevant articles or conference abstracts identified in the targeted literature search, none were included in the analysis because of a lack of patient-level VA data by age or female-only data (see Supplementary Table 2 in Additional File 1) [7, 25, 28, 49–56]. Because only 28 (3.9%) individuals from 3 (13.0%) studies had more than 1 BCVA measurement [20, 39, 48], the present study focused on cross-sectional data analysis. Data obtained from the literature were merged with BCVA measurements from the NIGHT study (320 participants, 613 eyes) [3, 26–29], creating a final data set of 1037 individuals and 1602 eyes (Table 1). Additionally, 37 females were excluded from our analysis. Overall, data from both eyes were available for 565 individuals.

**Table 1:** Baseline Characteristics

Parameter	Statistics
No. of individuals included	1037
From NIGHT study	320
Identified in literature	717
No. of eyes studied	1602
From NIGHT study	613
Identified in literature	989
Age, mean (SD), y	41.8 (16.2)
Age group, n (%)	
< 20 y	120 (12)
20 to < 40 y	315 (30)
40 to < 60 y	465 (45)
≥ 60 y	137 (13)

Mean (SD) age of individuals included in the analysis was 41.8 (16.2) years (Table 1). Mean (SD) age of participants in the NIGHT study (46.8 [13.4] years) was higher than mean (SD) age of individuals identified in the literature (39.5 [16.9] years). Mean (SD) BCVA for all eyes was 0.4 (0.6). Mean (SD) BCVA measurements for the BSE and WSE were 0.3 (0.5) and 0.7 (0.6), respectively (Table 2).

**Table 2:** BCVA Statistics

Characteristic	All eyes	Better-seeing eye	Worse-seeing eye
No. of eyes	1602	565	565
BCVA, mean (SD)	0.4 (0.6)	0.3 (0.5)	0.7 (0.6)
BCVA group, n (%)			
≤ 0.5	1164 (73)	490 (87)	315 (56)
> 0.5 to ≤1.3	340 (21)	57 (10)	197 (35)
> 1.3 to ≤1.7	23 (1)	0	21 (4)
> 1.7	75 (5)	18 (3)	32 (6)

*BCVA* best-corrected visual acuity. BCVA measured in logMAR units

### Rates of BCVA decline with age

**Fig. 1:**
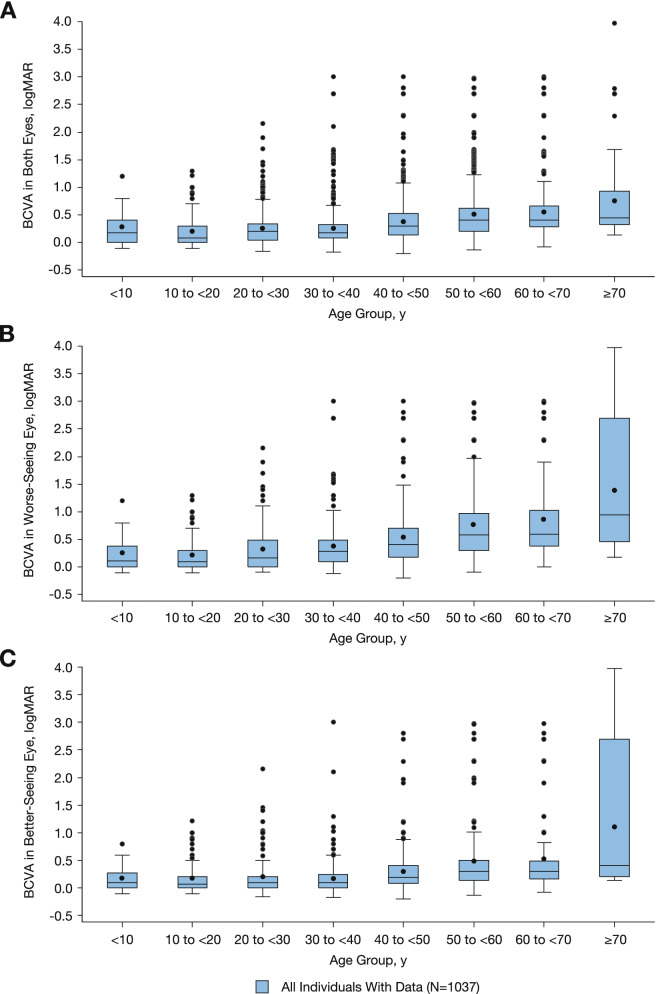
Mean BCVA (logMAR) by age groups with all available data in (**A**) both eyes, (**B**) worse-seeing eyes, and (**C**) better-seeing eyes. *BCVA *best-corrected visual acuity

**Fig. 2:**
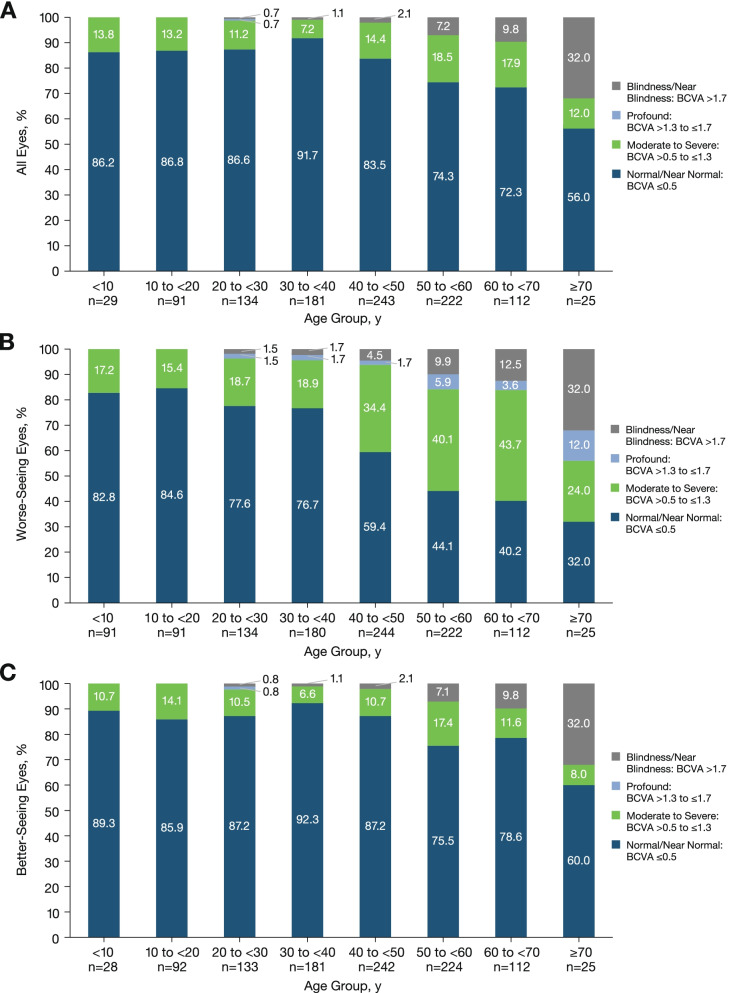
BCVA (logMAR) category distribution by age at baseline in (**A**) both eyes, (**B**) worse-seeing eyes, and (**C**) better-seeing eyes. *BCVA* best-corrected visual acuity

Mean BCVA was similar across age groups of individuals aged < 40 years and steadily declined with each age group thereafter for both eyes (Fig. 1A), the WSE (Fig. 1B), and the BSE (Fig. 1C). More than 80% of all eyes from individuals aged < 40 years had normal/near normal vision in both eyes (Fig. 2A). The proportion of those with normal vision steadily decreased in higher age groups; from all available baseline data, only 56% of all eyes from individuals aged ≥70 years had normal/near normal vision in both eyes (Fig. 2A). Similar trends were observed in the WSEs and BSEs in individuals aged < 40 years having normal/near normal vision at baseline (Fig. 2B and C). However, the proportion of WSEs with normal/near normal vision was notably lower than the proportion of BSEs with normal/near normal vision in individuals aged > 40 years at baseline. In individuals aged ≥70 years, 32% of WSEs had normal vision and 60% of BSEs had normal vision at baseline.

### Interocular BCVA decline

**Fig. 3:**
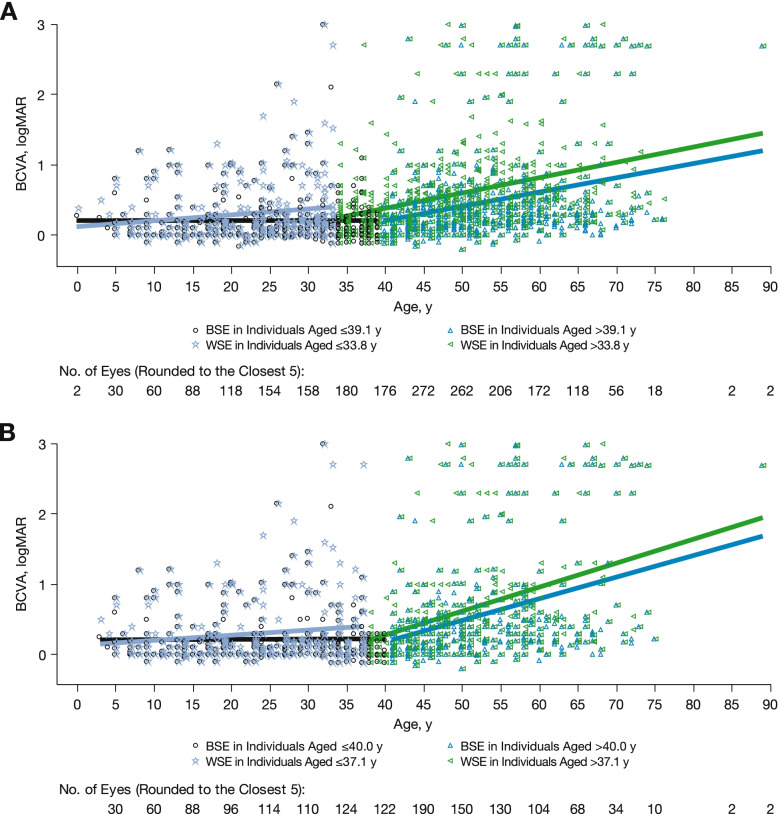
BCVA (logMAR) over time in WSE and BSE by age in (**A**) all individuals and (**B**) all individuals excluding those in the NIGHT study. Individual data points are represented, along with the segmented regression trendlines before and after the transition age in the BSE (black and blue lines, respectively) and WSE (lavender and green lines, respectively). *BCVA* best-corrected visual acuity, *BSE* better-seeing eye, *WSE* worse-seeing eye

The rate of BCVA decline was different between the BSE and WSE before the age of ~ 30 to ~ 40 years, with higher decline rates and an earlier decline transition in the WSE. A transition age cutoff for BCVA decline was identified with a segmented regression as 33.8 years in the WSE and 39.1 years in the BSE. The WSE and BSE showed similar BCVA trends; disease progression was initially higher in the WSE but was similar between eyes after the respective age cutoffs. In the WSE, the rate of BCVA decline was 0.0086 logMAR/year (95% CI, 0.0026 to 0.0146) in individuals aged ≤33.8 years and 0.0219 logMAR/year (95% CI, 0.0174 to 0.0264) in individuals aged > 33.8 years (Fig. 3A; Table 3). The BCVA decline rate in the BSE was lower than that in the WSE in individuals aged ≤39.1 years (0.00001 logMAR/year; 95% CI, − 0.0346 to 0.0346) but similar between eyes in individuals aged > 39.1 years (0.0203 logMAR/year; 95% CI, 0.0151 to 0.0257). This trend was similar when NIGHT study data were excluded (Fig. 3B; Table 3).

**Table 3:** Rates of BCVA Decline

Characteristic	BSE, logMAR/year (95% CI)	WSE, logMAR/year (95% CI)
All data		
≤ Age cutoff^a^	0.00001 (−0.0346, 0.0346)	0.0086 (0.0026, 0.0146)
> Age cutoff^a^	0.0203 (0.0151, 0.0257)	0.0219 (0.0174, 0.0264)
Excluding NIGHT		
≤ Age cutoff^b^	0.0010 (−0.0030, 0.0049)	0.0072 (0.0015, 0.0129)
> Age cutoff^b^	0.0310 (−0.0310, 0.0393)	0.0334 (0.0256, 0.0413)

*BCVA* best-corrected visual acuity, *BSE* better-seeing eye, *WSE* worse-seeing eye. BCVA measured in logMAR units

^a^Age cutoff points are 39.1 and 33.8 for BSE and WSE, respectively

^b^Age cutoff points are 40.0 and 37.1 for BSE and WSE, respectively

**Fig. 4:**
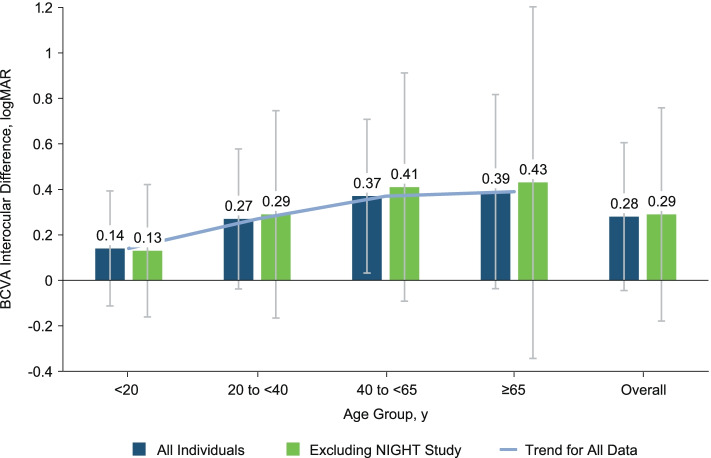
Interocular asymmetry based on mean difference in BCVA (logMAR) score between eyes by age group in all individuals (blue) and in all individuals excluding those in the NIGHT study (green). Overall trendline for all data (blue line) is shown. Error bars represent SD. *BCVA* best-corrected visual acuity

Evaluation of BCVA interocular symmetry revealed a moderate correlation of 0.60 (95% CI, 0.49 to 0.69) for individuals aged < 20 years; this decreased to 0.44 (95% CI, 0.40 to 0.49) for those aged 20 to < 40 years, 0.49 (95% CI, 0.40 to 0.57) for those aged 40 to < 65 years, and 0.51 (95% CI, 0.38 to 0.63) for those aged ≥65 years. Differences in BCVA between the BSE and WSE increased between age groups beginning at < 20 years to 40 to < 65 years (Fig. 4). This trend of increasing interocular asymmetry between age groups was not evident in age groups from 40 to < 65 years to ≥65 years (Fig. 4). When NIGHT study data were removed in a sensitivity analysis, no major change in BCVA interocular symmetry trends was observed.

## Discussion

Understanding the natural progression of BCVA decline in choroideremia can inform the interpretation of clinical studies and the design of future clinical studies. Furthermore, an understanding of vision decline based on age data may support ideal management strategies for future therapies. In the current analysis, we evaluated BCVA decline in a large data set and determined a differential rate of decline before and after the age of ~ 30 to ~ 40 years, with a younger transition age (i.e., earlier age of rapid BCVA decline onset) in the WSE compared with the BSE. Additionally, our results show increasing BCVA interocular asymmetry before the age of 40. Prior studies on BCVA decline in choroideremia have suggested a biphasic linear model of BCVA decline with a transition age of ~ 40 years [2, 4, 5, 7, 8, 22–24]. The current analysis includes data from the largest number of individuals with choroideremia used to evaluate the natural decline of BCVA, as well as an exploration of the differential rate of interocular BCVA decline.

In a recent meta-analysis of 1004 eyes by Shen et al., the BCVA decline rate was 0.24 letters/year before 39 years of age and 1.16 letters/year after 39 years of age [8]. Our analysis demonstrated similar results using BCVA decline by logMAR per year, with an increase in the rate of BCVA decline and proportion of individuals with moderate or worse BCVA occurring at ~ 40 years of age. Shen et al. also evaluated BCVA decline rates between eyes. They found that BCVA measurements of the right and left eye were moderately correlated. The authors also evaluated the time to impaired VA (i.e., BCVA ETDRS letter score of < 85) and found that the WSE and BSE dropped to impaired VA at comparable ages, whereas the WSE dropped to subnormal VA or legal blindness earlier than the BSE. In our analysis, we report BCVA decline in logMAR as it provides enhanced sensitivity for detecting interocular differences in VA and assessing VA in patients with severe vision loss [30, 57, 58]. We determined the rate of decline in BCVA using logMAR/year in both the WSE and BSE and found that the decline rate was higher in the WSE before the transition age but similar between eyes after ~ 40 years of age. Additionally, the transition to rapid BCVA decline occurred at an earlier age (33.8 years) in the WSE compared with the BSE (39.1 years).

The differential decline rate in BCVA between the 2 eyes may be related to differing interocular risk of foveal involvement. As the small central islands of the preserved areas of autofluorescence and ellipsoid zone generally shrink symmetrically toward the foveae as choroideremia progresses, the risk of foveal involvement increases bilaterally [27, 48, 59]; however, this risk may not be precisely the same for each eye. The fovea of the WSE could potentially become more affected compared with the fovea of the BSE, leading to differences in BCVA decline between the 2 eyes before the transition age. As choroideremia progresses with further bilateral foveal degeneration [45], BCVA of both eyes worsens [48], and differences in BCVA decline between the 2 eyes lessen after the transition age. Further research on the differential rate of BCVA decline observed in choroideremia is needed to understand the underlying mechanism.

Overall, there were similar findings from the full data set and from the sensitivity analysis excluding the NIGHT study. The NIGHT clinical study is the largest natural history study of choroideremia to date. The study included males with choroideremia with BCVA ranging from none/mild (BCVA ETDRS letter score of > 73) to severe (BCVA ETDRS letter score of < 34) [3, 27–29]. Our analyses utilized the baseline data from the NIGHT study along with published literature to include a large data set of individuals with choroideremia. The inclusion of a broader range of disease stages and various levels of BCVA severity in combination with standardized data collection from the NIGHT study demonstrate that the rate of BCVA decline observed in real-world cohorts is comparable with that observed in a clinical study setting. In addition to analyzing unilateral disease progression, we investigated in detail the differential progression rates and cutoff points between the BSE and WSE. In the recent article by Shen et al., bilateral progression was assessed broadly in the form of BSE and WSE Kaplan-Meier curves corresponding to the proportion of patients retaining above 35 and 85 letters. With addition of the NIGHT study, this analysis represents the largest data set in choroideremia.

The current analysis has some limitations: (1) longitudinal data were not available and are often difficult to obtain because of the rarity and slow-progressing nature of choroideremia (individual disease progression was therefore not identified); (2) females were not included because of the limited sample size and because disease progression profiles in females are reported to be different than those in males; (3) some individuals may have contributed the same data to more than one study (however, this is not expected to change the direction of the results as this was most likely a rare event); (4) not all individuals had bilateral data, reducing the sample size of this analysis; (5) some data points were extracted from graphs where one data point may have represented more than one individual or one individual may have contributed to the same graph more than once (though in our analysis, this would be considered as 2 independent data points); (6) inter-study heterogeneity may have affected overall results, although a sensitivity analysis excluding the NIGHT study was performed (e.g., our analysis included a study by van Schuppen et al. [4], which Shen et al. identified as a potential source for a large amount of heterogeneity in their analysis [8]); and (7) additional factors potentially contributing to VA decline, such as cataracts or refractive status, were not available for analyses as potential confounding variables.

## Conclusions

The results of this meta-analysis support differential rates of BCVA decline in each eye before and after an identified age cutoff. In 2 clinical studies of gene therapy for choroideremia, participant ages ranged from 32 to 72 years [19, 20]. Studies including participants aged above and below the age cutoff for BCVA decline should consider the differential decline rates when evaluating outcomes. Furthermore, identifying the age cutoff at which rapid BCVA decline begins presents an opportunity for early intervention in choroideremia to potentially prevent severe visual impairment. Additionally, natural history of interocular progression of visual impairment is important to understand as Phase 2 and 3 gene therapy studies have treated one eye, often the WSE, and used the BSE as a control [19, 20, 60]. Although the decision to treat both eyes involves considerations beyond BCVA, such as associated risks and effects on quality of life, an understanding of the differential BCVA decline between eyes may help physicians decide when and if to treat one or both eyes during clinical studies or with future therapies. With a novel gene therapy for choroideremia being investigated in a Phase 3 clinical study [60, 61], it is important for physicians to recognize the rate of BCVA decline to best inform study result interpretations and future treatment decisions.

## Supplementary Information

**Additional file 1: Supplementary Table 1**. Characteristics of Included Studies^a^. **Supplementary Table 2**. Articles Excluded From Analysis. **Supplementary Figure 1**. PRISMA flowchart.

## Data Availability

The data sets used and analyzed during the current study are available from the corresponding author on reasonable request.

## Abbreviations

BCVA: Best-corrected visual acuity
BSE: Better-seeing eye
ETDRS: Early Treatment Diabetic Retinopathy Study
NIGHT: Natural History of the Progression of Choroideremia
REP1: Rab escort protein 1
RP: Retinitis pigmentosa
VA: Visual acuity
WSE: Worse-seeing eye
