# Supplementary material for: Bilateral visual acuity decline in males with choroideremia: a pooled, cross-sectional meta-analysis
Source: BMC Ophthalmol. 2022 Jan 16;22:29. doi: 10.1186/s12886-022-02250-z (PMC8762852; doi:10.1186/s12886-022-02250-z)

Bilateral Visual Acuity Decline in Males With Choroideremia:

A Pooled, Cross-Sectional Meta-Analysis

Duygu Bozkaya,[1] Heng Zou[1] Cindy Lu,[1] Nicole W. Tsao,[1] Byron L. Lam[2]

[1]Biogen, Cambridge, MA, USA; [2]Bascom Palmer Eye Institute, Miami, FL, USA

Supplementary information.

Supplementary Table 1. Characteristics of Included Studies[a]

Study citation	Study type	Participants, n[b]	Eyes, n	Age, mean	Reported BCVA measurement	First eye BCVA, mean (logMAR)[c]	Second eye BCVA, mean (logMAR)[d]
Abbouda et al[34]	Retrospective analysis	36	70	38.3	logMAR	0.5	0.4
Di Iorio et al[22]	Retrospective analysis	51	102	38.0	logMAR	0.6	0.6
Dimopolous et al[35]	Prospective study	30	60	41.9	Snellen	0.4	0.5
Duncan et al[36]	Prospective study	13	26	34.6	Snellen	0.2	0.3
Fischer et al[37]	Phase 2 clinical trial	6	12	54.3	Snellen	0.5[e]	0.3[f]
Freund et al[2]	Retrospective analysis	110	110	38.0	logMAR	0.4[g]	NA
Genead et al[38]	Prospective study	2	4	49.5	logMAR	1.1	0.3
Han et al[39]	Retrospective analysis	10	20	41.7	logMAR	0.8	0.7
Hayakawa et al[47]	Retrospective analysis	15	29	37.0	logMAR	1.1	NA
Heon et al[5]	Retrospective analysis	60	60	36.4	logMAR	0.2[g]	NA
Jain et al[32]	Case series	7	14	24.2	Snellen	0.03	0.07
Lam et al[20]	Phase 2 clinical trial	6	12	51.0	ETDRS letter score	0.4[e]	0.2[f]
Lazow et al[40]	Prospective study	7	7	38.9	Snellen	0.4	0.1

Li et al[41]	Retrospective analysis	6	12	26.3	logMAR	0.4	0.4
Morgan et al[33]	Prospective study	57	85	39.0	Snellen	0.4	0.4
Renner et al[42]	Retrospective analysis	18	36	33.5	logMAR	0.7	0.8
Roberts et al[23]	Retrospective analysis	95	95	38.9	logMAR	0.3[g]	NA
Sanchez-Alcudia et al[43]	Retrospective analysis	34	67	27.1	logMAR	0.6	0.8
Sandberg et al[44]	Prospective study	6	6	54.0	Snellen	0.3[g]	NA
Seitz et al[45]	Retrospective analysis	69	69	43.0	logMAR	0.3[h]	NA
Simunovic et al[46]	Prospective study	71	71	44.6	logMAR	0.5	NA
van Schuppen et al[4]	Longitudinal prospective study	21	42	52.9	Snellen	1.0	1.4
Xue et al[48]	Phase 2 clinical trial	12	24	47.8	ETDRS letter score	0.3[e]	0.2[f]

ETDRS, Early Treatment Diabetic Retinopathy Study; NA, not applicable. [a]Mean age and BCVA were calculated on the basis of extracted data, including data extracted from figures. [b]Male participants only. [c]Denotes right eye, unless otherwise noted. [d]Denotes left eye, unless otherwise noted. [e]Study eye. [f]Control eye. [g]Better-seeing eye. [h]Eye not denoted.

Supplementary Table 2. Articles Excluded From Analysis

Article	Reason for exclusion
Abbouda et al[49]	VA by age not reported
Aleman et al[7]	Could not extract patient-level VA data
Aylward et al[50]	Conference abstract; patient-level VA data by age could not be extracted
Cunningham et al[51]	Conference abstract; patient-level VA data by age could not be extracted
Dysli et al[25]	Could not extract patient-level VA data
Freund et al[52]	Conference abstract; patient-level VA data by age could not be extracted
Jauregui et al[53]	Data from female patients only
Kahn et al[56]	VA by age not reported
Lam et al[28]	Conference abstract; patient-level VA data by age could not be extracted
Tracey-White et al[54]	Conference abstract; patient-level VA data by age could not be extracted
Zinkernagel et al[55]	Clinical trial report without patient-level VA data

VA, visual acuity.

Supplementary Figure 1. PRISMA flowchart. BCVA, best-corrected VA; VA, visual acuity.

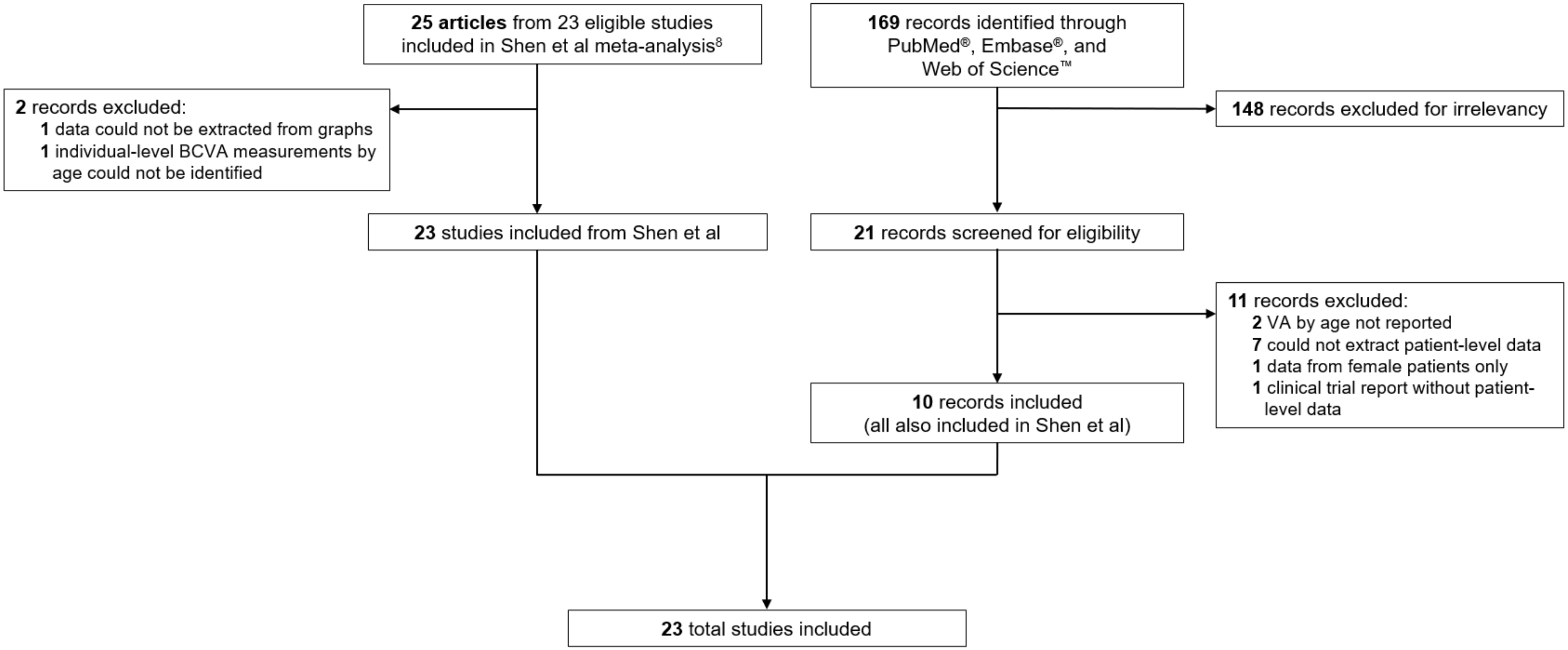

Supplement: Supplementary file 1 — Additional file 1: Supplementary Table 1. Characteristics of Included Studiesa. Supplementary Table 2. Articles Excluded From Analysis. Supplementary Figure 1. PRISMA flowchart. [file 12886_2022_2250_MOESM1_ESM.pdf]
